# A Late Presentation of a Fatal Disease: Juvenile Hemochromatosis

**DOI:** 10.1155/2013/875093

**Published:** 2013-09-11

**Authors:** Cynthia Cherfane, Pauline Lee, Leana Guerin, Kyle Brown

**Affiliations:** ^1^Department of Internal Medicine, Roy J. and Lucille A. Carver College of Medicine, University of Iowa, Iowa City, IA 52242, USA; ^2^Department of Molecular and Experimental Medicine, The Scripps Research Institute, La Jolla, CA 92037, USA; ^3^Department of Pathology, Roy J. and Lucille A. Carver College of Medicine, University of Iowa, Iowa City, IA 52242, USA; ^4^Iowa City Veterans Administration Medical Center, Iowa City, IA 52246, USA; ^5^Free Radical and Radiation Biology Program, Roy J. and Lucille A. Carver College of Medicine, University of Iowa, Iowa city, IA 52242, USA; ^6^Division of Gastroenterology and Hepatology, University of Iowa, 200 Hawkins Drive, 4553 JCP, Iowa City, IA 52242, USA

## Abstract

Juvenile hemochromatosis is a rare and severe form of hereditary hemochromatosis. We report the case of a 39-year-old female who presented with heart failure and cirrhosis from previously unrecognized juvenile hemochromatosis. This is the latest presentation described in the literature. An important clue to the diagnosis was a history of amenorrhea since the age of 20 that had never been investigated. The patient died of intractable heart failure two months after the initial presentation. Juvenile hemochromatosis should be suspected in a young patient with endocrine or cardiac manifestations. Early diagnosis is crucial since phlebotomy can improve the prognosis and delay or prevent progression to heart failure and cirrhosis.

## 1. Introduction

In 1932, Bezançon et al. described a case of a 20-year-old female with pigmentary cirrhosis, enlarged liver, infantilism, and multiple endocrine insufficiencies, who died of cardiac failure [1]. That was the first description of juvenile hemochromatosis (JH). In 1978, the disease was recognized as a distinct clinical entity with the description of a 26-year-old woman with heart failure, insulin-dependent diabetes, amenorrhea and hepatomegaly and a review of 52 previously reported cases of symptomatic idiopathic hemochromatosis [2, 3]. Since that time, there have been important discoveries that have clarified the molecular and genetic basis of this disorder, but limited progress has been achieved in improving its treatment, and JH is still often a fatal disease. We report the case of a woman who presented at the age of 39 with severe JH.

## 2. Case Presentation

A 39-year-old Caucasian woman presented with palpitations and a two-week history of progressive fatigue, shortness of breath, and abdominal pain with distension. Her past medical history was significant for hypothyroidism on thyroid replacement therapy and amenorrhea since the age of 20 that had not been evaluated. Family history was pertinent for a first cousin who had died suddenly of “cardiac arrest” at 20 years old. Her mother and father were both in good health. There was no family history of iron overload or liver disease. The patient was an unmarried teacher. She had never smoked, rarely drank alcohol, and had no history of illicit drug use. Her physical exam showed mild jaundice and an irregular cardiac rhythm. The abdomen was diffusely tender with hepatomegaly, no splenomegaly or evident ascites. An electrocardiogram showed atrial fibrillation with rapid ventricular response, and an echocardiogram showed globally decreased left ventricular systolic function with an estimated ejection fraction of 26%.

The patient was admitted to the cardiovascular intensive care unit where management of heart failure was initiated, her heart rate was controlled, and she was started on anticoagulation. Hematologic findings included a hemoglobin concentration of 13 g/dL, platelet count of 100 K/mm^3^, and leucocyte count of 9.7 K/mm^3^. Blood chemistries revealed the following results: total bilirubin 2.6 mg/dL, direct bilirubin 1.1 mg/dL, aspartate aminotransferase 1476 U/L, alanine aminotransferase 702 U/L, alkaline phosphatase 139 U/L, gamma-glutamyl transpeptidase 52, albumin 4.1 g/dL, and lactate dehydrogenase 968 U/L. Review of the peripheral blood smear showed no evidence of hemolysis. Coagulation studies showed a prothrombin time of 20 sec and an international normalized ratio (INR) of 2.1. Other relevant tests included thyroid-stimulating hormone 40 uIU/mL (normal 0.27–4.2), free thyroxine 1.05 ng/dL (normal 0.93–1.7), thyroid peroxidase autoantibody (anti-TPO) 1586 IU/mL (normal < 2), follicle-stimulating hormone 0.3 mIU/mL (normal 3.5–12.5 mIU/mL), and luteinizing hormone 0.1 mIU/mL (normal 2.4–12.6 mIU/mL). Abdominal ultrasound showed an enlarged liver with diffusely increased echogenicity, minimal ascites and a normal-sized spleen. Doppler findings were consistent with congestive heart failure.

**Figure 1:**
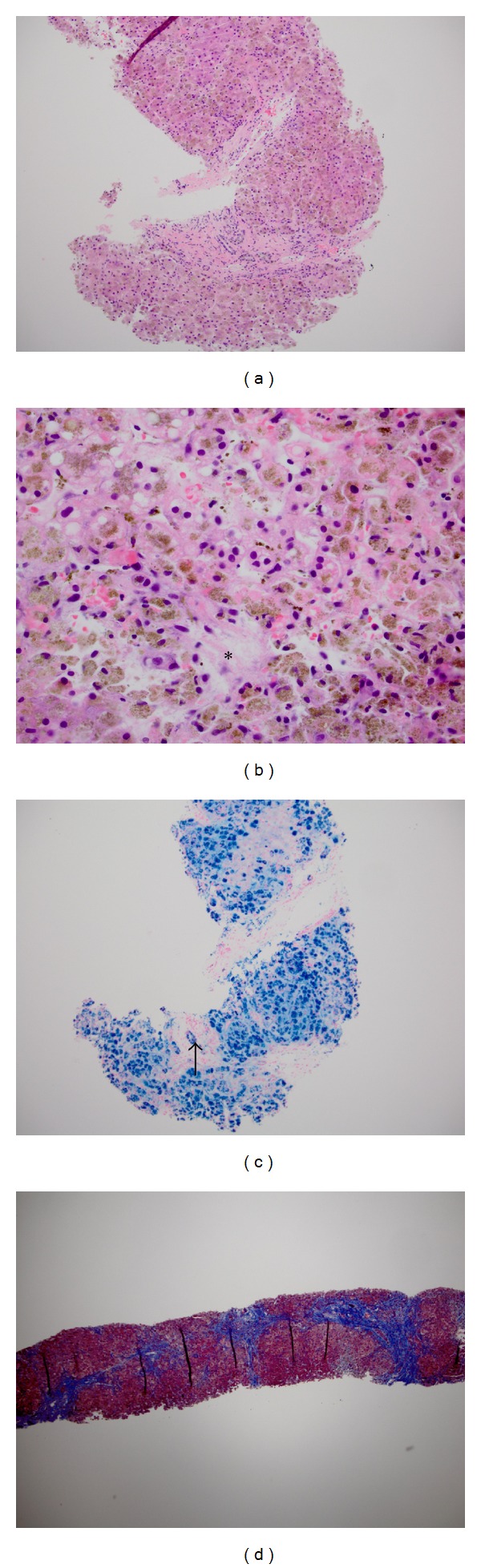
Liver biopsy from a young woman with juvenile hemochromatosis due to mutations in *HFE2*. (a) Iron appears as a brownish pigment in hepatocytes in this section stained with hematoxylin and eosin (10x magnification). (b) At higher power (40x magnification), the granular brown iron pigment is readily appreciated within hepatocytes. A small area of necrosis is indicated by the asterisk. (c) Perl's Prussian Blue stain confirms 4+ iron in hepatocytes as well as heavy iron deposition in the epithelium of a bile duct (arrow; 10x magnification). (d) Trichrome stain highlights the nodular contour of the hepatic parenchyma outlined by thick bands of connective tissue (4x magnification).

**Figure 2:**
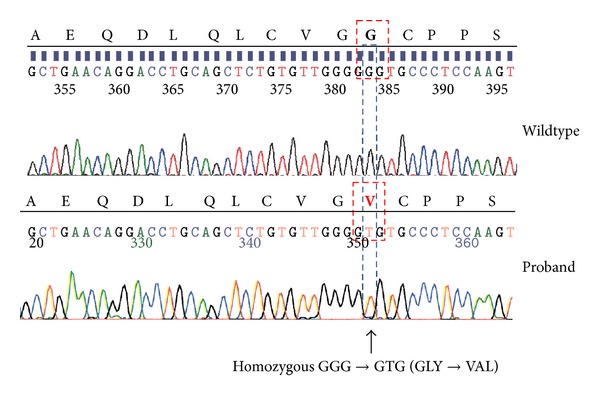
Sequencing demonstrating the hemojuvelin Gly320Val homozygous mutation.

Testing for viral hepatitis and autoimmune serologies was negative. Alpha 1-antitrypsin and ceruloplasmin levels were normal. However, iron studies were markedly abnormal with a transferrin saturation of 100% and ferritin of 116,070 ng/mL (normal 22–322 ng/mL). Transjugular liver biopsy showed cirrhosis with 4+ iron deposition in hepatocytes and in biliary epithelium, as well as focal centrilobular necrosis, likely due to congestion (Figure 1). *HFE *genotyping by the amplification refractory mutation system was negative for both C282Y and H63D mutations. JH was suspected and further testing was performed on a research basis. Amplification of the *HFE2 *gene by polymerase chain reaction with genotyping by direct sequencing disclosed that the patient was homozygous for the *HFE2 *Gly320Val mutation (Figure 2).

Once her heart rate and congestive heart failure were controlled, she began weekly phlebotomies. Erythropoietin was administered because of concomitant anemia, but she did not undergo chelation. One month after her presentation, except for persistent fatigue, her symptoms had resolved. The ferritin level had decreased to 7400 ng/mL. Unfortunately, two months later, after 8 phlebotomies, she was hospitalized for severe shortness of breath and hypotension. An echocardiogram showed worsening left ventricular function with ejection fraction of 10%. She was intubated and treated with vasopressors and an intra-aortic balloon pump to maintain her blood pressure. Antiarrhythmic medications were given for recurrent nonsustained ventricular tachycardia. Despite the aggressive medical management, she developed multiorgan failure and died five days after admission. The family declined an autopsy. As part of family screening, her mother had iron studies done, which were normal. Her father and half-brother declined screening with iron studies.

## 3. Discussion

JH is an autosomal recessive form of hereditary hemochromatosis (HH) characterized by severe iron overload presenting in early adulthood. In contrast to the more common form of HH associated with *HFE *mutations, in which iron overload-related disease predominates in males, men and women are equally affected in JH. This may be explained in part by amenorrhea resulting from central hypogonadism in these young women [2]. In a review of 37 cases from Italy, the average age of presentation of JH was 23.6 years [3, 4]. Our patient was 39, which is the oldest age of presentation of JH among the cases described in the literature. However, her long history of amenorrhea indicates that central hypogonadism predated the other manifestations of JH. Reported incidences of hypogonadism and cardiomyopathy at presentation of JH are 94.6% and 43.2%, respectively, and are the earliest and most frequent manifestations of JH [3]. It is hypothesized that this is secondary to a high susceptibility of the heart and the pituitary to iron at a younger age. The presence of cirrhosis at initial presentation is less common in JH (27%); its presence in our patient may relate to her older age and longer duration of disease [2–4]. Given the increased level of TSH and anti-TPO, her hypothyroidism was most likely secondary to Hashimoto's disease rather than pituitary infiltration. Hashimoto's thyroiditis has been reported in two other JH cases [5]. The level of ferritin at presentation in our case was much higher than the mean level reported in the literature (3217 ng/mL) and likely reflected liver injury from congestive heart failure superimposed on iron overload [3].

Patients presenting with HH have mutations in the *HFE *gene in 85–90% of cases. The remaining patients have mutations in other genes [6–9]. Many of these have JH, which is also known as hemochromatosis type 2. This is a rare form of HH described worldwide, although its exact prevalence remains unknown [3]. JH type 2A is associated with mutations in the *HFE2 *gene on chromosome 1q (previously known as *HJV*) [10–12] and accounts for 90% of cases of JH. The *HFE2 *Gly320Val mutation seen in our patient is one of 47 mutations described in the *HFE2 *gene and is the most frequently reported mutation [13, 14]. Compared to the low penetrance of the *HFE *mutations, all patients who have a homozygous mutation of the *HFE2 *gene will manifest the disease clinically, although clinical expression may be variable [3, 15]. 

The *HFE2 *gene encodes the hemojuvelin protein, which upregulates the expression of hepcidin, a hormone produced by the liver that plays a central role in the regulation of iron homeostasis. The target of hepcidin's action is ferroportin, an iron exporter expressed on the cell membranes of enterocytes and macrophages. Binding of hepcidin to ferroportin causes internalization and degradation of the latter protein, thereby blocking egress of iron from the cell [16]. Hepcidin expression is modulated by multiple physiologic stimuli acting through a variety of signaling pathways. Under normal circumstances, hepcidin increases in response to iron, a feedback mechanism that prevents iron overload. Upregulation of hepcidin expression by iron is mediated by a pathway involving bone morphogenetic protein 6 (BMP6). Hemojuvelin is a BMP6 coreceptor on hepatocytes whose presence is required for induction of hepcidin expression by BMP6 [17]. The Gly320Val mutation causes a defect in the proteolytic processing of hemojuvelin that prevents targeting the protein to the plasma membrane and thus abrogates BMP6 signaling [18]. As a consequence, hepcidin expression no longer responds appropriately to iron status and iron overload ensues. A similar phenotype results from mutations in the gene encoding hepcidin itself, which accounts for the remaining 10% of cases of JH (type 2B) [19]. In addition, defective hepcidin responses and/or hepcidin resistance are common to the other forms of hereditary hemochromatosis as well as secondary iron overload [14]. 

JH is a life-threatening disease; however morbidity and mortality can be prevented or attenuated if diagnosed and treated early. Iron studies with ferritin and transferrin saturation should be done when the diagnosis is suspected. In those with increased iron parameters, *HFE *genotyping should be obtained. 

Liver biopsy or magnetic resonance imaging should be considered to confirm iron overload in patients with elevated iron studies but without compatible *HFE *mutations [20]. The *HFE2 *Gly320V is the molecular test of choice in a young adult suspected to have JH [21–23]; however, genetic analysis for non-*HFE *forms of HH is not routinely available, leading to a clinical diagnosis in most cases. Phlebotomy is still considered the treatment of choice for JH. If initiated early, it can slow the progression of the disease and improve the prognosis [24–26]. In our patient, an early investigation of her amenorrhea could have yielded the diagnosis, allowing the initiation of phlebotomy and, potentially, a better outcome. Relative contraindications to phlebotomy include anemia, acute heart failure, and hemodynamic instability [26, 27]. After our patient was stabilized hemodynamically, she underwent weekly phlebotomies with improvement in her symptoms and decrease in her ferritin levels. She was not started on iron chelation therapy, but in retrospect, this should have been considered. There are limited but encouraging data on the use of iron chelation therapy in JH patients who present with decompensated heart failure, with improvement in cardiac function, blood counts, liver function, and ferritin levels after weeks to months of therapy. Single regimens with IV deferoxamine and combined regimens with deferiprone and deferoxamine have been used [27–30]. Erythropoietin has been suggested in anemic patients as a means of maintaining regular phlebotomies and increasing iron mobilization [31]. Heart transplant and placement of a pacemaker are two other potential treatments when heart failure and arrhythmias are present. Our patient may also have benefited from hormone replacement therapy for hypogonadotropic hypogonadism and prevention of osteoporosis, which is a frequent complication of JH [32].

In conclusion, our case illustrates the poor prognosis of JH, in which delayed diagnosis and treatment resulted in severe cardiomyopathy and death in the fourth decade from heart failure and/or arrhythmia [9, 27]. In retrospect, an aggressive regimen of chelation may have yielded a better outcome. Early suspicion in young patients with endocrine and/or cardiac disease and diagnosis with iron studies and genotype is crucial since early treatment with phlebotomy can change the natural history of the disease. With the better understanding of the disease at the molecular and biological levels, new therapies could be promising such as new chelating agents, exogenous transferrin, exogenous hepcidin, hepcidin analogues, and hepcidin signaling agonists [33]. Unfortunately, clinical trials to study the role of these new therapies and iron chelation therapy in JH are limited by the rarity of the disease.
